# Driven to Death

**Published:** 1870-12

**Authors:** 


### DRIVEN TO DEATH.

If you visit the chief business men of New York any day, and invite them to go to the country for a short time, or to take a sail on the water, or a drive to the Central Park for a few hours, in nine times out of ten the answer will be, "It is impossible to-day." Another will tell you, "I have not taken a regular dinner in ten days." A third will declare, "I have been up until twelve o'clock every night for a week." And so you may go the rounds of fifty men, each will have his answer, not a soul will go to-day, but every one will express the hope that soon the press of business will be over, and they will be but too happy to take a rest. One of the first bankers in New York, who has been so busy for ten years in making two millions of dollars, although a clever fellow, and a handsome man, has "not had time" to look around for a wife. Many days, from nine to four, he is on his feet without a single moment's intermission. And yet he is as cheerful as a lark, and as lively as a young kitten; he is kept up by the stimulus of making money—but one of these days soon, without a change, Wall Street will be shocked with the intelligence, "Died to-day." Nobody had ever heard of his being sick; he was "on the street yesterday." "He seemed as well as he ever was two hours ago." These are the histories of our most successful business men, repeated every year and every month. But the misfortune is, that their death is attributed to every other than the right cause. The immediate agent or cause of their sudden taking off has the credit, or the blame—it is apoplexy, or paralysis, or pneumonia, or hemorrhage, or something else, leaving out of sight the most important practical fact, that their systems were attacked by these ailments because they had exhausted their stamina, all their vitality, all their life, and there did not remain in the system the power to resist the slightest attack of any disease. The first essential of every man engaged in large or responsible business is the greatest amount of early, regular, uninterrupted sleep at night; the second necessity of the business man is regular, leisure eating; without these, early disablements, or premature death, are inevitable.

